# Incidental cardiac findings on computed tomography imaging of the thorax

**DOI:** 10.1186/1756-0500-3-326

**Published:** 2010-12-03

**Authors:** Paul WX Foley, Ali Hamaad, Hossam El-Gendi, Francisco Leyva

**Affiliations:** 1 Department of Cardiology, University of Birmingham, Good Hope Hospital, Sutton Coldfield, West Midlands, B72 1JW, UK; 2 Centre for Cardiovascular Sciences, Queen Elizabeth Hospital, University of Birmingham, UK

## Abstract

**Background:**

Investigation of pulmonary pathology with computed tomography also allows visualisation of the heart and major vessels. We sought to explore whether clinically relevant cardiac pathology could be identified on computed tomography pulmonary angiograms (CTPA) requested for the exclusion of pulmonary embolism (PE). 100 consecutive CT contrast-enhanced pulmonary angiograms carried out for exclusion of PE at a single centre were assessed retrospectively by two cardiologists.

**Findings:**

Evidence of PE was reported in 5% of scans. Incidental cardiac findings included: aortic wall calcification (54%), coronary calcification (46%), cardiomegaly (41%), atrial dilatation (18%), mitral annulus calcification (15%), right ventricular dilatation (11%), aortic dilatation (8%) and right ventricular thrombus (1%). Apart from 3 (3%) reports describing cardiomegaly, no other cardiac findings were described in radiologists' reports. Other reported pulmonary abnormalities included: lung nodules (14%), lobar collapse/consolidation (8%), pleural effusion (2%), lobar collapse/consolidation (8%), emphysema (6%) and pleural calcification (4%).

**Conclusions:**

CTPAs requested for the exclusion of PE have a high yield of cardiac abnormalities. Although these abnormalities may not have implications for acute clinical management, they may, nevertheless, be important in long-term care.

## Introduction

Computed tomography pulmonary angiography (CTPA) has become a first line investigation for suspected pulmonary embolism (PE) [1-3]. Although primarily geared towards visualisation of the lungs, currently available CTPA also provides images of the heart. Incidental lung findings, such as benign and malignant nodules, can be found in patients undergoing cardiovascular magnetic resonance (CMR) [4] and cardiac CT [5-8]. We sought to determine the frequency cardiac abnormalities in patients undergoing CTPA for suspected PE.

## Methods

One hundred consecutive patients aged 64 (19) (mean [SD]) underwent CTPA in the period April to July 2007. The scans, acquired on a non-gated conventional 16-slice CT scanner (Philips Brilliance scanner, city, country) using bolus tracking, were analysed retrospectively and independently by two cardiologists (AH, PWXF). Any disagreements in findings were referred to a third cardiologist (HEG) with Level 2 cardiac CT accreditation. These reports were compared with previously issued radiological reports. Both atrial and ventricular dimensions were made in the transverse plane at the mid-ventricular level.

### Statistical analysis

Absolute numbers of findings for each type of pathology was converted into a percentage of the total number of scans analysed.

## Results

### Pulmonary abnormalities

As shown in Table 1, 5% of scans revealed evidence of PE. Additional pulmonary abnormalities included: lung nodules (14%), lobar collapse/consolidation (8%), emphysema (6%), pleural effusion (2%) and pleural calcification (4%). This amounted to 39% of scans showing pulmonary abnormalities.

**Table 1:** Summary of incidental cardiac findings on 100 CT scans requested for the diagnosis of possible pulmonary embolism (%)

Pulmonary findings	39
Lung nodules	14
Lobar collapse/consolidation	8
Emphysema	6
Pulmonary embolism	5
Pleural disease	4
Pleural effusion	2
**Cardiac findings**	
Aortic wall calcification	54
Coronary calcification	46
Cardiomegaly (CTR > 1:2)	41
Bi-ventricular dilatation	16
Left ventricular dilatation	14
Right ventricular dilation	11
Atrial dilatation	18
Mitral annulus calcification	15
Aortic dilatation	8
Intracardiac thrombus	1

### Cardiac abnormalities

**Figure 1:**
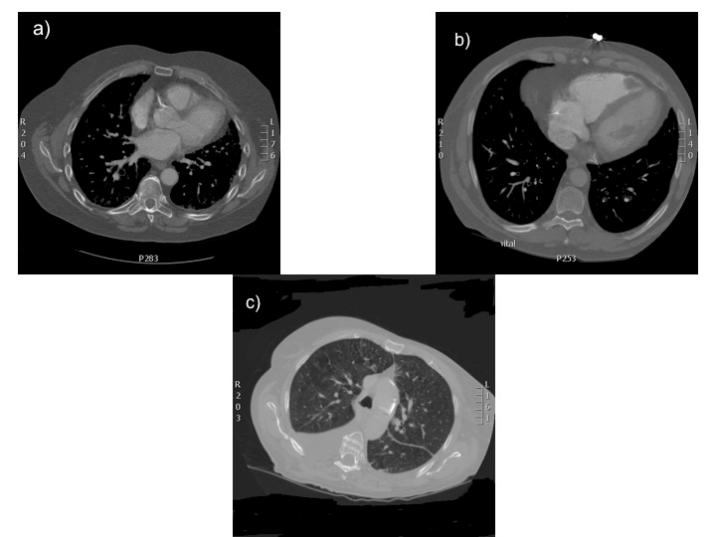
**Examples of incidental findings observed on CT pulmonary angiograms**. Figure 1a: coronary calcification, Figure 1b: right ventricular mural thrombus. Figure 1c: aortic wall calcification.

According to the retrospective report from cardiologists, 78% of scans had at least one incidental cardiac finding (Table 1). According to the previous radiological reports, only 3 contained a reference to a cardiac finding namely, 3 cases of cardiomegaly. Figure 1 illustrates some examples of incidental cardiac findings.

## Discussion

Computed tomography pulmonary angiography has become a routine investigation for the investigation of acute PE. The British Thoracic Society and European Society of Cardiology guidelines recommend the use of CTPA as the initial investigation method of choice in non-massive PE [9]. In this consecutive series of patients admitted as an emergency for the exclusion of PE, only 5% of CTPAs yielded evidence of PE. Importantly, however, cardiac abnormalities were evident in 78% of scans. Prominent amongst these were coronary and aortic wall calcification, and cardiomegaly. Less frequent findings included atrial dilatation and calcification of the aortic valve and/or mitral annulus.

Although incidental cardiac findings may not be relevant to the immediate clinical management of patients with suspected PE, they may nevertheless influence long-term clinical management. For example, coronary and aortic wall calcification are markers of atherosclerosis [10,11]. According to several studies of asymptomatic individuals with subclinical atherosclerosis, treatment with statins, [12-14] and aspirin [15-17] improve atherosclerotic burden and prolongs survival. Reporting of these cardiac findings may therefore influence primary prevention of atherosclerotic events. Cardiomegaly, on the other hand, is a late feature of left ventricular dysfunction and heart failure [18], both of which carry a very poor prognosis [19]. Inclusion of such incidental findings on thoracic CTPA reports could therefore trigger further cardiac investigations and treatment. Failure to report cardiovascular pathology in CT scans has been recently highlighted in a study of aortic root dilatation in the Veteran Affairs Health Care System [20].

We conclude that thoracic CT scans frequently yield evidence of incidental cardiovascular pathology. Whilst these findings may not be relevant to immediate clinical management, they may well be clinically relevant in the long-term. Radiologists' reports of thoracic CT scans should therefore include cardiac findings.

## Key messages

1. Incidental cardiac findings of significant pathology are common on CT scans of the thorax.

2. Reporting of cardiac abnormalities is important as potentially management may be changed as a result

3. Cardiac abnormalities are more common than pulmonary emboli on CT scans

4. Cardiac failure is associated with cardiomegaly. The presence of cardiomegaly should prompt further cardiac investigation.

5. Incidental cardiac findings are usually not reported.

## Competing interests

The authors declare that they have no competing interests.

## Authors' contributions

AH and PWXF analysed the scans together and wrote the manuscript. HEG arbitrated where there was dispute in the findings and contributed to the manuscript. FL inspired the study and as senior author re-wrote parts of the manuscript. All authors have read and approved the final manuscript.
